# Elevated CXCL1 expression in breast cancer stroma predicts poor prognosis and is inversely associated with expression of TGF-β signaling proteins

**DOI:** 10.1186/1471-2407-14-781

**Published:** 2014-10-24

**Authors:** An Zou, Diana Lambert, Henry Yeh, Ken Yasukawa, Fariba Behbod, Fang Fan, Nikki Cheng

**Affiliations:** Department of Pathology and Laboratory Medicine, University of Kansas Medical Center, Kansas City, KS 66160 USA; Department of Biostatistics, University of Kansas Medical Center, Kansas City, KS 66160 USA; Department of Biology, Beloit College, Beloit, WI 53511 USA

**Keywords:** CXCL1, Chemokine, Stroma, Fibroblast, Breast Cancer, TGF-beta, SMAD2, SMAD3, Prognosis

## Abstract

**Background:**

CXCL1 is a chemotactic cytokine shown to regulate breast cancer progression and chemo-resistance. However, the prognostic significance of CXCL1 expression in breast cancer has not been fully characterized. Fibroblasts are important cellular components of the breast tumor microenvironment, and recent studies indicate that this cell type is a potential source of CXCL1 expression in breast tumors. The goal of this study was to further characterize the expression patterns of CXCL1 in breast cancer stroma, determine the prognostic significance of stromal CXCL1 expression, and identify factors affecting stromal CXCL1 expression.

**Methods:**

Stromal CXCL1 protein expression was analyzed in 54 normal and 83 breast carcinomas by immunohistochemistry staining. RNA expression of CXCL1 in breast cancer stroma was analyzed through data mining in http://www.Oncomine.org. The relationships between CXCL1 expression and prognostic factors were analyzed by univariate analysis. Co-immunofluorescence staining for CXCL1, α-Smooth Muscle Actin (α-SMA) and Fibroblast Specific Protein 1 (FSP1) expression was performed to analyze expression of CXCL1 in fibroblasts. By candidate profiling, the TGF-β signaling pathway was identified as a regulator of CXCL1 expression in fibroblasts. Expression of TGF-β and SMAD gene products were analyzed by immunohistochemistry and data mining analysis. The relationships between stromal CXCL1 and TGF-β signaling components were analyzed by univariate analysis. Carcinoma associated fibroblasts isolated from MMTV-PyVmT mammary tumors were treated with recombinant TGF-β and analyzed for CXCL1 promoter activity by luciferase assay, and protein secretion by ELISA.

**Results:**

Elevated CXCL1 expression in breast cancer stroma correlated with tumor grade, disease recurrence and decreased patient survival. By co-immunofluorescence staining, CXCL1 expression overlapped with expression of α-SMA and FSP1 proteins. Expression of stromal CXCL1 protein expression inversely correlated with expression of TGF-β signaling components. Treatment of fibroblasts with TGF-β suppressed CXCL1 secretion and promoter activity.

**Conclusions:**

Increased CXCL1 expression in breast cancer stroma correlates with poor patient prognosis. Furthermore, CXCL1 expression is localized to α-SMA and FSP1 positive fibroblasts, and is negatively regulated by TGF-β signaling. These studies indicate that decreased TGF-β signaling in carcinoma associated fibroblasts enhances CXCL1 expression in fibroblasts, which could contribute to breast cancer progression.

**Electronic supplementary material:**

The online version of this article (doi:10.1186/1471-2407-14-781) contains supplementary material, which is available to authorized users.

## Background

Breast cancer remains the most common form of cancer diagnosed in women in the US and the world, with over 1.3 million new cases annually [1, 2]. 80% of all invasive breast cancers in the US are diagnosed as invasive ductal carcinoma (IDC). Current treatments for IDC include radiation, chemotherapy, hormone therapy and targeted HER2 therapy [3–5]. Yet, up to 56% of patients with stage III breast cancer still experience disease recurrence. Disease recurrence for patients with late stage breast cancer is often accompanied by distant metastasis, contributing to an 80% mortality rate [6, 7]. Treatment effectiveness is complicated by the presence of reactive stroma, which is associated with tumor invasiveness and drug resistance [8–11]. In order to tailor treatments more effectively to the individual patient, it is important to define clearly the breast tumor stroma at a molecular level, which will enable us to identify biomarkers that will more accurately predict patient responsiveness to treatments.

Fibroblasts are a key cellular component in breast stroma, normally activated during mammary gland development to regulate ductal branching and morphogenesis [12, 13]. De-regulation of fibroblast growth and activity is associated with breast cancer. Carcinoma-associated fibroblasts (CAFs) are commonly identified by their spindle cell morphology and expression of mesenchymal markers including Fibroblast Specific Protein 1 (FSP1), alpha Smooth Muscle Actin (α − SMA), and Fibroblast Activating Protein (FAP) [14, 15]. Accumulation of CAFs strongly correlates with tumor grade and poor patient prognosis [16–18]. Co-transplantation studies and transgenic mouse studies have demonstrated that CAFs enhance breast tumor growth and invasion [19–21]. Conversely, co-transplantation of normal fibroblasts with breast cancer cells inhibits cellular invasiveness and inhibits tumor progression [22]. These studies indicate that fibroblasts may enhance or inhibit breast cancer progression dependent on the tissue of origin.

Recent studies demonstrate the importance of CAFs in chemo-resistance. Fibroblasts are more resistant to chemotherapy than cancer cells, including melanoma and squamous cell carcinoma [23]. In animal models, Doxorubicin treatment results in increased CAF secretion of growth factors and cytokines involved in the development of drug resistant prostate and colorectal cancers [24, 25]. Targeting FAP expressing CAFs in animal models has been shown to inhibit growth of invasive tumors and enhance chemo-sensitivity to Doxorubicin in colon and breast cancers [26, 27]. Yet, the use of FAP inhibitors has not been successful in clinical trials [28, 29]. This result may be due in part to the complex identity of CAFs. Fibroblasts are not a uniform population of cells. One type of CAF in breast cancer is the myofibroblast, which expresses α − SMA [30, 31]. Another type of breast CAF expresses FSP1 but not α − SMA [32]. Furthermore, fibroblasts may be derived from different origins including embryonic mesenchyme, endothelial cells, macrophages and cancer cells [15]. These studies indicate the presence of different populations of CAFs. Currently, the molecular signals that identify tumor-promoting fibroblasts remain poorly understood.

Emerging studies indicate an important clinical significance for chemokine expression in cancer stroma. Chemokines are a family of small soluble proteins (8-10 kda) that regulate angiogenesis and immune cell recruitment during inflammation and cancer [33–35]. Chemokines bind to seven transmembrane spanning receptors which couple to G proteins and activate signaling pathways involved with cell migration and differentiation. As a large family of molecules, chemokines are categorized into distinct families: C, C-C, C-X-C, and CX3C, in which a conserved cysteine motif may also include an amino acid (X) in their NH2 terminal domain. The C-X-C chemokine family is currently comprised of 17 ligands, which bind promiscuously to 7 chemokine receptors (CXCR1-7). A conserved glutamic acid-leucine-arginine (ELR) motif has been detected in a small subset of C-X-C chemokines (CXCL1, 2, 3, 5, 8), which is important for stimulating angiogenesis and regulating recruitment of neutrophils [36, 37]. Up-regulated expression of ELR positive chemokines have been detected in various cancers, associated with increased angiogenesis and immune cell recruitment. CXCL3 is up-regulated in prostate cancer [38] while CXCL5 has been detected in lung and liver cancers [39]. Increased expression of CXCL1 has been reported in multiple tumor types including prostate cancer, gastric cancer, renal cell carcinoma and melanoma [40, 41]. These studies indicate aberrant expression of C-X-C chemokines in cancer.

Recent reports have implicated a role for CXCL1 in breast cancer. Increased CXCL1 protein expression was associated with increased tumor growth and pulmonary metastasis of MDA-MB-231 breast cancer cells grafted in the mammary fat pads of nude mice [42]. Increased CXCL1 protein expression has been reported in HER2 positive metastatic breast cancer [43]. Increased plasma levels of CXCL1 protein are associated with decreased survival of patients with metastatic disease [44]. Similarly, increased tumoral expression of CXCL1 RNA is associated with metastatic disease, correlating with tumor grade and decreased survival of patients with ER-α positive breast cancer [45]. These studies demonstrate a clinical significance for CXCL1 expression in breast cancer.

Previous studies have reported positive RNA expression of CXCL1, CXCL3, CXCL5, CXCL6 and CXCL8 in stromal cells including: blood-circulating cells, fibroblasts and endothelial cells [45]. These studies indicate that expression of binding ligands to CXCR2 is not restricted to epithelial cells. However, no further studies have been conducted to examine the prognostic significance of RNA expression of CXCR2 binding ligands in the breast cancer stroma, or examine their protein expression patterns in the stroma. Biomarker expression patterns in the stroma and epithelium can have vastly different relationships to known prognostic factors and clinical outcomes [46]. Given the importance of CXCL1 expression in breast cancer, the goal of this study was to: characterize further the expression patterns of CXCL1 in breast cancer stroma, determine the prognostic significance of stromal CXCL1 expression and identify factors affecting stromal CXCL1 expression. We used a combination of data-mining analysis and immunohistochemistry staining of patient samples to investigate the RNA and protein expression patterns of CXCL1 in the breast stroma. Our studies indicated that patient samples expressed high levels of CXCL1 RNA and protein in breast cancer stroma, correlating with tumor grade. CXCL1 RNA expression levels were significantly associated with tumor recurrence and decreased patient survival. CXCL1 protein expression co-localized to FSP1 and α-SMA positive cells, indicating that CXCL1 is expressed in more than one population of CAFs. Increased CXCL1 in CAFs correlated with decreased TGF-β expression. Immunostaining analysis of breast tumor tissues indicated that increased CXCL1 expression inversely correlated with expression of TGF-β, phospho-SMAD2 and phospho-SMAD3. Treatment of cultured CAFs with TGF-β suppressed CXCL1 secretion and promoter activity. In summary, these studies indicate a prognostic significance for CXCL1 expression in breast cancer stroma, show that CXCL1 is localized to multiple fibroblast populations, and is negatively regulated by TGF-β signaling.

## Methods

### Patient samples used for immunohistochemistry analysis

Samples were collected from commercial (US Biomax Inc) and institutional resources from the University of Kansas Medical Center. Characteristics of patients from both datasets are summarized (Table 1). When the datasets were combined, the median age of normal patients was 48.6 years, 51 years for DCIS patients and 50.5 years for IDC patients.

**Table 1:** **Characteristics of breast ductal carcinoma samples from US Biomax and the BRCF core combined**

Prognostic factor	No. of DCIS cases (percentage of total)	No. of IDC cases (percentage of total)
**Histologic grade**		
1	2 (9%)	10 (18%)
2	7 (32%)	24 (41%)
3	12 (59%)	24 (41%)
**Tumor size**		
*>2 cm*	16 (70%)	10 (36%)
*<2cm*	7 (30%)	18 (64%)
**BCL2**		
*negative*	4 (27%)	8 (42%)
*positive*	11 (73%)	11 (57%)
**P53**		
*negative*	7 (38%)	9 (36%)
*positive*	11 (62%)	16 (64%)
**Ki67**		
***>50%***	3 (16%)	5 (22%)
***<50%***	16 (84%)	18 (78%)
**ER**		
***negative***	7 (38%)	9 (36%)
***positive***	11 (62%)	16 (64%)
**PR**		
***negative***	7 (38%)	11 (47%)
***positive***	11 (62%)	13 (53%)
**HER2**		
***negative***	0	7 (32%)
***positive***	18 (100%)	15 (68%)
**EGFR**		
***negative***	7 (38%)	10 (50%)
***positive***	11 (62%)	10 (50%)
**Lymph node**		
**status**	NA	
***negative***		12 (52%)
***positive***		11 (48%)

#### US biomax samples

Tissue microarrays (TMA) containing de-identified cores of 18 normal and 26 invasive breast ductal carcinoma samples were obtained from US Biomax (cat. nos. 8032 and 241). Normal breast tissue samples came from adjacent tissues of breast cancer patients. The breast samples were collected from patients originating in South Korea and China. Normal women had a median age of 43 years and women with IDC had a median age of 44.6 years.

#### Biospecimen Repository Core Facility (BCRF)

Patient samples of normal, Ductal Carcinoma In Situ (DCIS) and IDC were obtained from the BRCF, an IRB approved facility at the University of Kansas Medical Center. Out of the 36 normal samples collected from the BCRF, 13 samples were collected from adjacent tissues of breast cancer patients, and 23 samples were collected from patients undergoing reduction mammoplasty. Tumor samples were collected from Caucasian women who were diagnosed with primary breast ductal carcinoma, and had not been treated with radiation or chemotherapy before sample collection. Fourteen normal, 5 DCIS and 18 IDC specimens were obtained as individual paraffin blocks. Tissue microarrays were generated from an additional 22 normal, 20 DCIS and 14 IDC specimens. Normal women had a median age of 51.5 years and women with IDC had a median age of 51 years.

Pathology reports included information on clinical diagnosis, and information on tumor grade, tumor size, lymph node status, biomarker expression and age. DCIS samples were graded according to the Van Nuys System. IDC samples were graded according to the Scarff-Bloom and Richardson system. Intensity of staining or percentage of positive cells were reported for BCL2, p53, ER, PR, Her2 and EGFR biomarkers, and are summarized as positive or negative. As the samples were collected within the last 4 years, no follow-up data was available. Prognostic factor data that was present in more than 55% of the pathology reports were reported. Tumor grade and age were combined from both US Biomax and the BCRF (Table 1).

### Immunohistochemistry staining

CXCL1 protein expression was examined on patient samples obtained from US Biomax and the BRCF core. Expression of TGF-β, phospho-SMAD2 and phospho-SMAD3 proteins was primarily analyzed on patient samples obtained from the BRCF core. Tissue sections (5 microns) were de-waxed and rehydrated in PBS. Sections were subjected to antigen retrieval in 10 mM sodium citrate buffer pH 6.0 for 10 minutes at 100°C and washed in PBS. Endogenous peroxidases were quenched in PBS containing 3% H_2_0_2_ and 10% methanol for 30 minutes. After rinsing in PBS, samples were blocked in PBS containing 5% rabbit serum and incubated with antibodies (1:100) to CXCL1 (cat. no. 1374, Santa Cruz Biotechnology), TGF-β (cat. no. MAB 240, R&D Systems), phospho-SMAD2 (Ser465/467) (cat. no. 3101, Cell Signaling Technologies), or phospho-SMAD3 (Ser 423/425) (cat. no. C25A9, Cell Signaling Technologies) overnight at 4°C. Samples were washed in PBS and incubated with secondary goat biotinylated antibodies (1:500) (cat. no. BA-5000, Vector Labs), conjugated with streptavidin peroxidase (cat. no. PK-4000, Vector Labs) and incubated with 3,3′-Diaminobenzidine (DAB) substrate (cat. no. K346711, Dako). Sections were counterstained with Harris’s hematoxylin for 5 minutes, dehydrated and mounted with Cytoseal.

### Quantification of immunohistochemistry staining

Immunohistochemistry staining was imaged at 10× magnification using a Motic AE 31 microscope with Infinity 2-1c color digital camera. Four fields were captured for each at 10× magnification. To analyze biomarker expression in stromal tissues, we adapted methods described in previous studies [47–49]. Images were first imported into Adobe Photoshop. Hue and saturation of images were normalized using Auto-Contrast. Tumor epithelium was distinguished from stroma by differences in nuclear and cellular morphology, and tissue architecture. Using the lasso tool, epithelial tissues were selected and cropped out from the image, leaving the stromal tissues behind. These stromal tissues were labeled as “total stromal area.” DAB chromogen staining (brown) was selected using the Magic Wand Tool in the Color Range Window, with a specificity range of 66. The selected pixels were copied and pasted into a new window and saved as a separate file. DAB positive images were opened in Image J and converted to greyscale. Background pixels resulting from luminosity of bright-field images were removed by threshold analysis. Images were then subject to particle analysis. Positive DAB staining and total stromal areas were expressed as particle area values of arbitrary units. Positive DAB values were normalized to total stromal values.

### Immunofluorescence staining

Normal or breast cancer sections were de-paraffinized and treated with sodium citrate as described for immunohistochemistry. Sections were permeabilized in PBS containing 10% Methanol for 30 minutes, washed in PBS and blocked for 1 hour with PBS containing 3% fetal bovine serum. Mouse IgGs were blocked using the M.O.M kit (cat. no. BMK-2202, Vector Labs) according to commercial protocol. For co-immunofluorescence staining of CXCL1 and FSP1, sections were incubated with goat polyclonal antibodies to CXCL1 at a 1:100 dilution (cat. no. 1374, Santa Cruz Biotechnology), and with rabbit polyclonal antibodies to FSP1 (pre-diluted solution cat. no. 27597, Abcam) in PBS/3% FBS overnight. For co-staining of CXCL1 and α-SMA, sections were incubated with antibodies overnight at 4°C, to CXCL1 at a 1:100 dilution, and mouse monoclonal antibodies to α-SMA at a 1:100 dilution (cat. no. ab134813, Abcam). Sections were then washed in PBS and incubated with the following secondary antibodies at a 1:500 dilution in blocking buffer for 1 hour: anti-goat-alexa-488 to detect CXCL1 expression, anti-mouse-alexa-568 to detect α-SMA, or anti-rabbit-alexa-488 to detect FSP1 expression. Sections were washed in PBS and countered with DAPI. Slides were mounted in Anti-Fade (cat. no. P36935, Invitrogen). Fluorescence images were taken at 20× magnification using the Motic AE-31 microscope.

### RNA expression analysis

RNA expression values in breast stromal samples were obtained from the microarray database in http://www.Oncomine.org, characterized by Finak et al. in previous studies [9, 50]. Briefly, tissue samples were collected from 53 patients with invasive breast carcinoma, of which 50 were diagnosed as IDC. Stromal samples were collected by laser capture micro-dissection and hybridized to microarrays. Six normal samples were obtained from adjacent tissues of breast cancer patients. Patient samples included follow-up data, including information on recurrence and poor survival outcome. With a 5 year follow-up, 8 patients exhibited no recurrence and 11 patients exhibited recurrence. There were no data on the remaining 34 patients with IDC. Poor survival outcome was defined as patients who died from disease at the time of follow-up. 43 patients were alive without disease, 4 patients were alive with disease, 3 patients died of disease and 1 patient died of other causes. The Finak database provided as Log_2_ median RNA expression values and prognostic information, including age, tumor grade and tumor size. The database did not include information on which cases were invasive lobular carcinoma, and were therefore included in the analysis.

### Cell culture

Primary mammary carcinoma-associated fibroblasts (CAFs) were isolated from MMTV-PyVmT transgenic mice [51] at 12-16 weeks of age. Primary normal mammary tissue associated fibroblasts (NAFs) were isolated from wild-type C57/BL6 mice at 12-16 weeks of age. FspKO fibroblasts were isolated from FspKO knockout mice as described [49]. Fibroblast cell lines were generated by spontaneous immortalization of primary mammary fibroblasts, as described [49]. Primary human fibroblasts were isolated from patient samples from reduction mammoplasty or invasive ductal carcinoma from the BRCF, using methods described [52]. Primary cells were cultured on 10-cm dishes coated with rat tail collagen I. All cells were cultured in Dulbecco’s modified Eagle medium (DMEM) containing 10% fetal bovine serum (FBS) (cat. no. FR-0500-A, Atlas Biological), 2 mM L-glutamine (cat. no. 25-005-CI, Cellgro) and 100 I.U/ml of penicillin/100 μg/ml of streptomycin (cat. no. 10-080, Cellgro).

### ELISA

Cells were seeded in a 24-well plate at a density of 20,000 cells for 24 hours. Conditioned medium was generated by incubating cells in 500 μl Opti-MEM media for 24 hours, and then centrifuged to eliminate cell debris. One hundred microliters of conditioned media, which were generated from indicated cell lines, were subjected to TGF-β ELISA (cat. no. DY1679, R&D Systems) or CXCL1 ELISA (cat. no. 250-11, Peprotech). Samples were analyzed according to manufacturer’s protocol. Reactions were catalyzed using a tetramethylbenzidine substrate (cat. no. 34028, Thermo Scientific) according to manufacturer’s protocol. The reaction was stopped with 1 M HCl, and absorbance was read at *A*_450nm_ using a 1420 multi-label plate reader (VICTOR3 TM V, PerkinElmer). All the samples were analyzed in triplicate.

### Luciferase assay

Cells were seeded in 6-cm dishes at a density of 150,000 cells for 24 hours, and then co-transfected with 8 μg of firefly luciferase plasmids (PGL3.luc.CXCL1) and 400 ng of Renilla luciferase plasmids (plasmid 12177: plS2, Addgene) using 8.4 μl Lipofectamine LTX and 15 μl Plus reagents according to manufacturer’s protocol (Invitrogen, life technologies). After 24 hours, cells were allowed to recover in Opti-MEM media containing 10% FBS for 24 hours. Cells were re-seeded in a 24-well plate at a density of 20,000 cells for 24 hours followed by incubation in serum free Opti-MEM media overnight. Cells were treated with Opti-MEM media containing 10% FBS in the presence or absence of 5 ng/ml of TGF-β for 24 hours. Cell lysates were analyzed using the Dual-Luciferase Reporter Assay system (cat. no. E1910, Promega) according to manufacturer’s instructions. Cells were rinsed twice with PBS, lysed in 100 μl of passive lysis buffer for 15 min at room temperature on a shaker. Cell lysates were sonicated for 10 seconds on ice, followed by centrifugation to eliminate cell debris. Twenty microliters of lysates were assayed in triplicate in 96 well opaque plates (cat. no. 3912, Corning Costar) using the Veritas Microplate Luminometer (model number 9100-202, Turner BioSystems).

### Ethics and consent statements

The tissues collected for these studies were categorized under the “Exemption Class,” according to regulations set forth by the Human Research Protection Program (ethics committee) at the University of Kansas Medical Center (#080193). Ethics approval was also obtained from the Human Research Protection Program at the University of Kansas Medical Center for the isolation of primary human fibroblasts from patient biospecimens. Written informed consent for tissue collection was obtained by the BRCF. Tissue samples were de-identified by the BRCF prior to distribution to the investigators. Existing medical records were used in compliance with the regulations of the University of Kansas Medical Center. These regulations are aligned with the World Medical Association Declaration of Helsinki.

Ethics approval was obtained from the Institutional Animal Care and Use Committee at the University of Kansas Medical Center for the isolation of PyVmT mammary carcinoma cells and fibroblasts.

### Statistical analysis

In vitro experiments were performed in a minimum of triplicate. Data are expressed as Mean ± SEM. Statistical analysis for in vitro experiments was determined using two-tailed *t* tests or one way ANOVAs with Bonferonni’s post-test comparisons in Graphpad Software. Statistical Significance was determined as *p* ≤0.05.

Sample populations did not fit a Gaussian distribution and were observed to be uneven. The uneven sample populations were due to two factors. Not all prognostic factors were consistently reported on pathology reports provided with the biospecimens. In addition, some tissue samples on tissue microarrays did not adhere to the slide during staining. Therefore, RNA and protein expression values and their relationships with prognostic factors were analyzed using non-parametric methods. Level of biomarker expression between two groups was analyzed by Log-rank Test or Wilcoxon two-sample test. Level of biomarker expression among more than 2 groups was analyzed by Kruskall-Wallis test with Dunn’s post-*hoc* comparison between groups. Spearman rank correlation was used to analyze the relationship between biomarker expression and prognostic factors that were expressed as continuous variables. The Wilcoxon Two-Sample Test was used to analyze the relationship between biomarker expression and prognostic factors (such as tumor grade), which were expressed as discrete variables. Statistical significance was determined by confidence levels >95% and *p* <0.05.

## Results

### Expression of CXCL1 RNA and protein are elevated in breast cancer stroma

**Figure 1:**
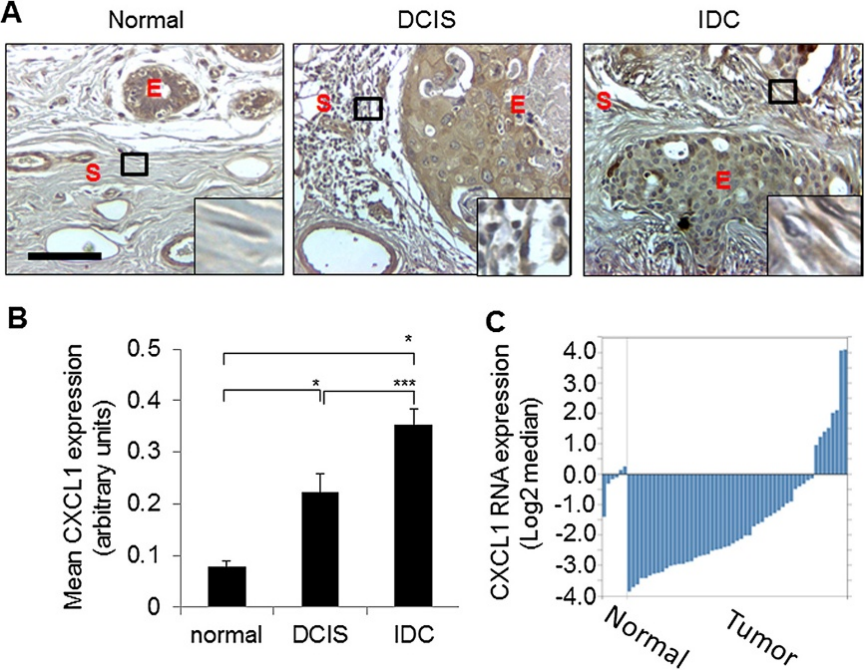
**CXCL1 expression is upregulated in the stroma of breast ductal carcinomas. A**. CXCL1 expression was analyzed by immunohistochemistry staining in normal (*n* =54), DCIS (*n* =25) or IDC (*n* =58) tissues. S = Stroma, E = epithelium. Magnified insets show representative CXCL1 staining in stroma. Scale bar = 50 microns. **B**. Staining in stroma was quantified by Image J analysis. Statistical analysis was determined by Kruskall-Wallis test with Dunn’s post-*hoc* comparison. *p ≤0.001 ***p ≥0.05. Values are expressed as Mean ± SEM. **C**. CXCL1 RNA expression values were obtained from the Finak microarray database (Oncomine.org) and analyzed for expression among patient samples.

To determine the significance of CXCL1 expression in breast stroma, we analyzed the protein and RNA levels of CXCL1 in breast cancer stroma. Using immunohistochemistry approaches, we first analyzed CXCL1 protein expression patterns in tissues from normal tissues, pre-invasive lesions known as Ductal Carcinoma in Situ (DCIS) [3, 53], and IDC tissues. CXCL1 protein expression in the stroma was quantified by software analysis, a method that was shown to be more reproducible, more consistent and less biased, compared to manual scoring [47, 48]. Consistent with previous studies [45, 54], CXCL1 was expressed in the tumor epithelium and in the stroma (Figure 1A). By immunohistochemistry, 87% of normal samples and 100% of DCIS and IDC samples were positive for CXCL1 protein expression. CXCL1 expression was significantly higher in DCIS and IDC stroma compared to normal stroma (Figure 1B). Expression of CXCL1 in IDC stroma was higher than DCIS stroma; however the difference was not significant. To determine RNA expression patterns of stromal CXCL1, we analyzed the microarray dataset on invasive breast cancer stroma generated by Finak et al., which was comprised of 53 cases of invasive breast carcinoma and 6 cases of normal breast samples [9]. We observed that 33% of normal samples (*n* =2), and 24% of IDC samples (*n* =12) were positive for CXCL1 RNA expression (Figure 1C). In the subset of positive samples, mean intensity of expression in the normal sample group was 0.19 ± 0.07 (Mean ± SD) compared to 2.18 ± 1.23 in IDC stroma. Overall, these data indicate higher intensity of CXCL1 expression in breast cancer stroma compared to normal breast stroma.

Breast ductal carcinomas often exhibit different architectural patterns, leading to the classification of different histological subtypes, which may have prognostic significance. The comedo subtype is associated with increased invasiveness, while rarer subtypes including cribribiform, mucinous and papillary tumors are associated with a good prognosis [55, 56]. In these studies, we examined for differences in expression of stromal CXCL1 among the different subtypes of breast cancer. The majority of tumor samples were classified as ductal carcinoma- not otherwise specified (NOS), consistent with the trend of the larger patient population [55]. Additional samples were classified as mixed solid/cribribiform, solid or comedo subtype. While stromal CXCL1 was positively expressed in all groups, there were no significant differences in expression among the subtypes in either DCIS or IDC patient samples (Additional file 1: Figure S1 and Additional file 2: Figure S2). We were unable to draw conclusions on mucinous, micropapillary and micropapillary/solid tumors with only one sample provided in each group, which reflected the rarity of these subtypes. In these studies, we can only conclude that CXCL1 is expressed in the stroma of breast ductal carcinomas of multiple histologic subtypes.

### Associations between stromal CXCL1 expression with risk factors, prognostic factors and patient outcomes

**Figure 2:**
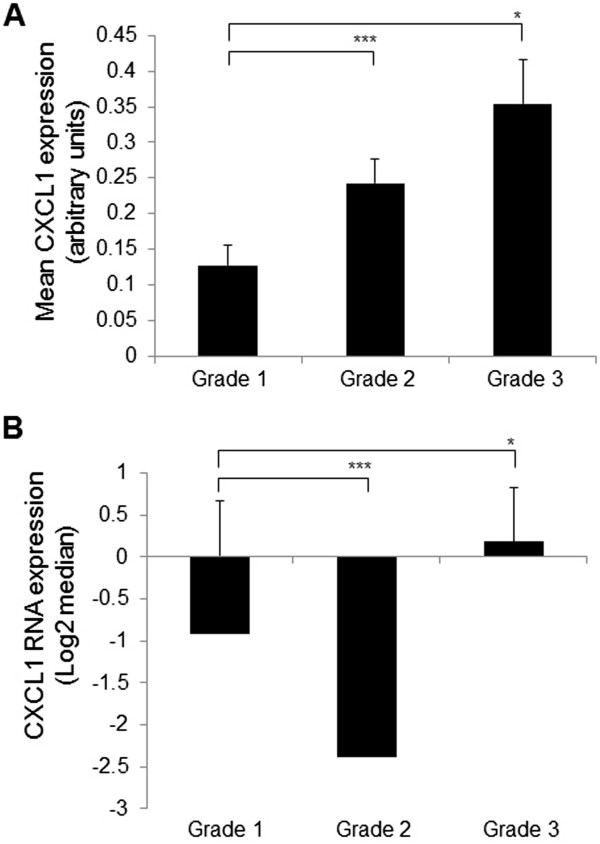
**Stromal CXCL1 expression is associated with tumor grade. A**. Stromal CXCL1 protein expression was analyzed for association with tumor grade of IDC by Kruskall-Wallis tests, followed by Dunn’s post-*hoc* comparison. **B**. CXCL1 RNA expression values were analyzed for association with tumor grade. Statistical analyses were performed using Wilcoxon Two-Sample Tests. Statistical significance was determined by *p* <0.05. *p ≤0.001 ***p ≥0.05. Values expressed as Mean ± SEM.

We first examined for differences between the US Biomax and BCRF datasets that would potentially affect stromal CXCL1 expression. In particular, we examined for associations with age and ethnicity, which were the risk factors consistently provided by both datasets. The median age of IDC patients was 46 for the US Biomax dataset, and 51 for the BCRF dataset. Despite the differences in age, there were no statistically significant associations between stromal CXCL1 and age in either dataset, as determined by Spearman Correlation Analysis (Additional file 3: Table S1). Samples from US Biomax dataset originated from patients in South Korea and China while the BCRF samples came primarily from Causasian women. Despite these ethnic differences, there were no significant differences in patterns of stromal CXCL1 between the two datasets (Additional file 4: Figure S3). These data indicate that stromal CXCL1 expression is not significantly associated with age or ethnicity, and that there are no observable differences in stromal CXCL1 between the two datasets. We then analyzed for associations between stromal CXCL1 and established prognostic factors by combining both datasets. There were no significant associations between protein expression of CXCL1 among DCIS and IDC stromal tissues with: tumor size, BCL2 expression, P53 status, ER, PR, HER2 status, EGFR expression, lymph node status, Ki67 expression or age, which is also recognized as a prognostic factor [57, 58] (Table 2). Increased stromal CXCL1 protein expression did not significantly correlate with grade of DCIS (Additional file 5: Figure S4), but was significantly associated with IDC tumor grade (Figure 2A). Furthermore, CXCL1 RNA expression was significantly associated with high grade tumors (Figure 2B). There was no significant association with age or tumor size (Table 3). In summary, these data indicate a statistically significant association between stromal CXCL1 expression and tumor grade.

**Table 2:** **Relationship between known prognostic factors and CXCL1 protein expression in breast cancer stroma**

Factor	r	95% CI	p-value	n
Age	0.12	-0.03 to 0.40	0.08	79
tumor size	0.18	-0.11 to 0.44	0.19	51
BCL2	-0.06	-0.40 to 0.29	0.72	34
P53	0.08	-0.23 to 0.38	0.60	43
Ki67	0.25	-0.07 to 0.51	0.12	41
ER	-0.07	-0.37 to 0.24	0.70	42
PR	0.10	-0.22 to 0.40	0.51	42
HER2	0.14	-0.19 to 0.44	0.40	40
EGFR	0.07	-0.26 to 0.39	0.63	38
No. of lymph node metastases	0.17	-0.14 to 0.45	0.28	23

Association between CXCL1 protein expression and commonly used prognostic markers was determined in DCIS and IDC stromal tissues using Spearman Correlation analysis. Significance was determined by *p*<0.05. r= correlation coefficient.

**Table 3:** **Relationship between known prognostic factors and CXCL1 RNA expression in breast cancer stroma**

Prognostic factor	r	95% CI	***p***-value
Age	-0.13	-0.41 to 0.17	0.38
Tumor size	-0.004	-0.31 to 0.30	0.97

Associations were determined using Spearman Correlation analysis of data obtained from the Finak microarray database. Significance determined by *p*<0.05.

r= correlation coefficient. N=53.

**Figure 3:**
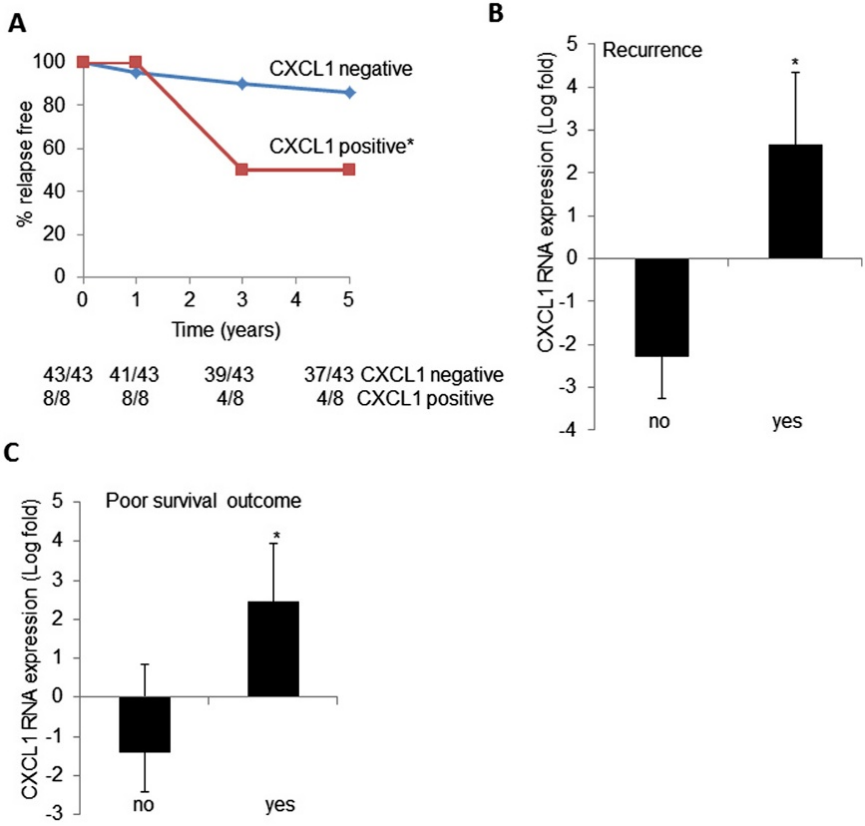
**Increased CXCL1 RNA expression in breast cancer stroma is associated with poor prognosis.** CXCL1 RNA expression values were obtained from the Finak microarray database, and analyzed for the following. **A**. The percentage of patients negative or positive for CXCL1 expression exhibiting tumor recurrence over time. The fractions below the graph depict recurrence-free patients over the total number of CXL1 negative or CXCL1 positive patients. **B**. Associations with overall tumor recurrence after 5 years. **C**. Associations with decreased survival after 5 years**.** Statistical analysis was performed using the Log-rank Test **(A)** or Wilcoxon Two-Sample Test (**B** and **C**). Mean ± SD. Statistical significance was determined by *p* <0.05. *p ≤0.001, ***p≥0.05.

Patient samples used for immunohistochemistry analysis were collected within the last 4 years, and did not include outcome data. However, we were able to analyze for associations between stromal CXCL1 RNA levels and tumor recurrence and poor survival in Oncomine using the Finak database. We quantified the number of recurrence-free patients that were negative or positive for CXCL1 expression. A total of 10/53 or 19% of patients experienced tumor recurrence, consistent with 5 year follow-up studies showing that 11 to 19.3% of patients with IDC experience disease recurrence [59, 60]. The percentage of recurrence-free patients in the CXCL1 positive group significantly decreased over time, from 1 to 5 years (Figure 3A). We analyzed the cohort of patient samples, in which tumor recurrence was measured after 5 years of treatment, and found a significant correlation between increased CXCL1 RNA expression in breast cancer stroma and increased tumor recurrence (Figure 3B). These data indicate a significant association between stromal CXCL1 RNA expression and disease recurrence. To determine whether the increased tumor recurrence was related to changes in patient survival, we analyzed the patient cohort for relationships between stromal CXCL1 RNA and survival. Patients with a poor survival outcome showed significantly higher levels of expression (Figure 3C). In summary, these data that increased CXCL1 expression is associated with increased recurrence and decreased survival.

### Elevated expression of CXCL1 in stromal derived fibroblasts is associated with decreased TGF-β signaling

**Figure 4:**
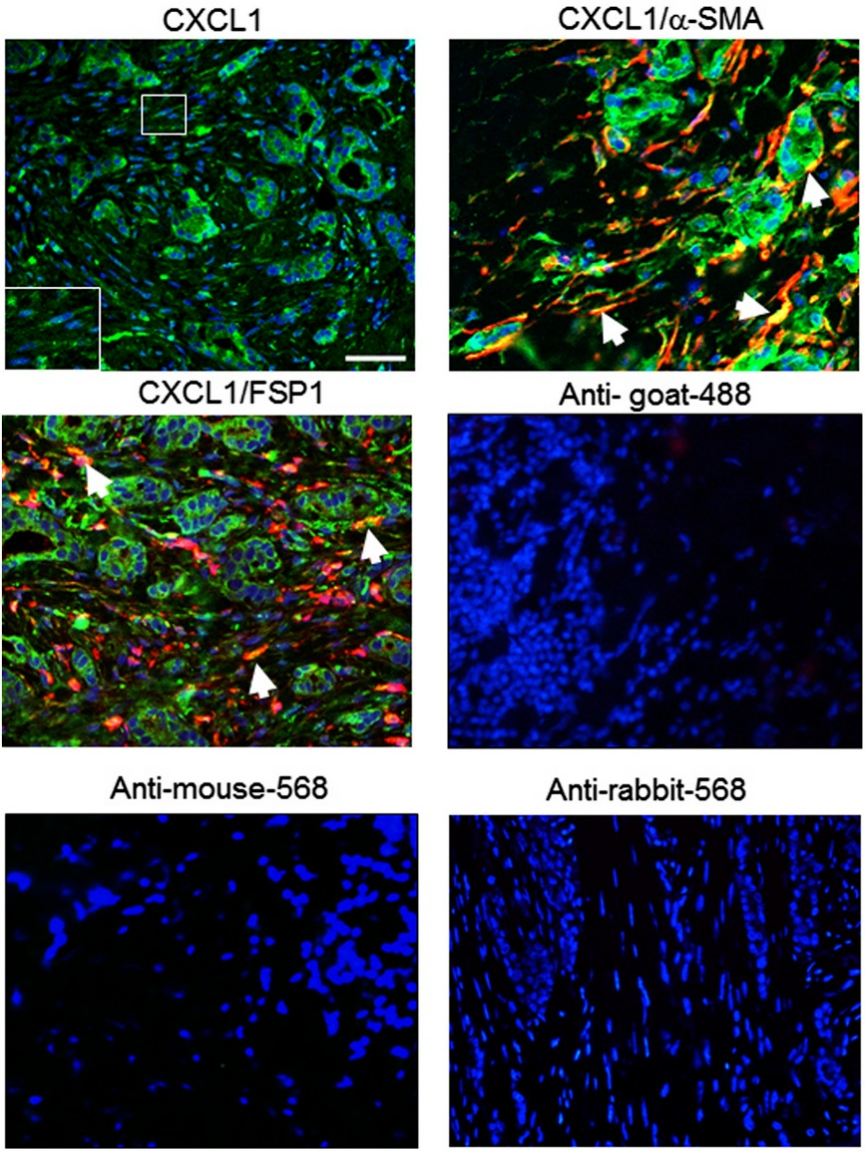
**CXCL1 co-localizes with α-SMA and FSP1 positive stroma.** Patient samples of breast ductal carcinoma were co-immunofluorescence stained for expression of CXCL1 (green) and α-SMA or FSP1 (red). Representative samples of CXCL1, α-SMA and FSP1 are shown. Sections were counterstained with DAPI. Secondary antibody only controls are shown: anti-goat-alexa-488 for CXCL1, anti-mouse-alexa-568 for α-SMA and anti-rabbit-alexa-568 for FSP1. Arrows and inset point to positive staining in fibroblastic cells. Scale bar =100 microns.

CXCL1 has been shown to be induced in fibroblasts by melanoma cells [61]. Breast CAFs were also positive for CXCL1 RNA expression [45]. These studies indicate that cancer associated fibroblasts are a potential source of CXCL1 expression. Fibroblasts in breast cancer stroma show non-overlapping expression of α- SMA and FSP1, indicating the presence of different subsets of fibroblasts [32]. To determine whether CXCL1 was expressed in particular fibroblast subsets in breast cancer, we performed co-immunofluorescence staining for CXCL1 expression with α-SMA or FSP1. Expression of CXCL1 was positive in the tumor epithelium and stroma, consistent with DAB expression patterns. We observed that CXCL1 overlapped with both α-SMA and FSP1 expressing cells (Figure 4). Some α-SMA and FSP1 positive cells did not express CXCL1, possibly reflecting differences in gene expression activity of these fibroblasts. In summary, these data indicate CXCL1 is expressed in both α-SMA and FSP1 positive fibroblasts in breast cancer stroma.

**Figure 5:**
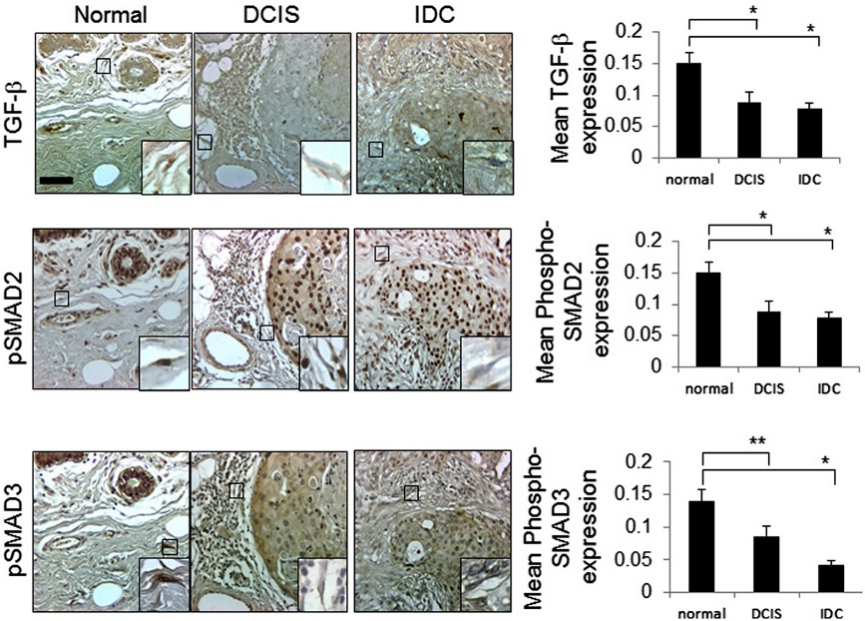
**Expression patterns of CXCL1 and TGF-β signaling proteins in breast cancer stroma.** Adjacent sections of normal breast (n=54) and invasive breast carcinoma (n=57) on TMAs were subject to immunohistochemistry staining for TGF-β, phosphorylated SMAD2 and phosphorylated SMAD3 proteins. Magnified insets show representative staining in fibroblastic cells. Expression was quantified by Image J, arbitrary units. Scale bar =50 microns. Statistical analysis was performed using Kruskall-Wallis tests, followed by Dunn’s post-*hoc* comparison. Statistical significance was determined by *p* <0.05. *p ≤0.001, **p ≤0.05. Values are expressed as Mean ± SEM.

We observed stromal CXCL1 expression was independent of many known prognostic factors (Table 2), and that CXCL1 expression was localized to CAFs. Therefore, we analyzed for molecular factors affecting CXCL1 expression in fibroblasts. Transforming Growth Factor Beta (TGF-β) signaling modulates cell proliferation and induces production of growth factors, angiogenic factors, extracellular matrix proteins and proteases in fibroblasts. These processes are vital for mammary ductal branching and morphogenesis during mammary gland development [62]. As an important regulator of fibroblast activity, the TGF-β pathway was a strong candidate. Therefore, we compared the protein expression patterns of stromal CXCL1 with TGF-β, and expression of phosphorylated SMAD2 and phosphorylated SMAD3, key downstream effector proteins [62, 63]. Decreased expression of TGF-β, phosphorylated SMAD2 and phosphorylated SMAD3 proteins were observed in DCIS and IDC stromal tissues, compared to normal stroma (Figure 5). Positive expression of stromal CXCL1 was inversely correlated with expression of TGF-β, phosphorylated SMAD2 and phosphorylated SMAD3 proteins (Table 4). These data indicate an inverse correlation between stromal CXCL1 protein expression and expression of TGF-β related proteins. We also analyzed the RNA expression patterns of CXCL1 and TGF-β related genes including *TGFB1, TGFBR2, SMAD2* and *SMAD3*. By Spearman correlation analysis, no significant associations were detected between stromal *CXCL1* RNA expression and expression of *TGFB1*, *SMAD3* or *TGFBR2* genes. *CXCL1* expression positively correlated with *SMAD2* gene expression (Table 5). In summary, these data indicate a negative correlation between stromal CXCL1 protein expression and expression of TGF-β signaling components, and a positive correlation between RNA expression of *CXCL1* and *SMAD2.*

**Table 4:** **Protein expression of TGF-β signaling components inversely correlate with CXCL1 expression in breast stroma**

Signaling component	r	95% CI	***p***-value	n
TGF-β	-0.33	-0.52 to -0.09	0.01	69
p-Smad2	-0.25	-0.44 to -0.027	0.02	80
p-Smad3	-0.32	-0.50 to -0.10	<0.01	81

Spearman Correlation used to determine the association between expression of CXCL1 and expression of TGF-β signaling components in normal and breast cancer stroma. Normal stroma refers to samples from both reduction mammoplasty and adjacent breast tissue.

Significance was determined by p<0.05. r= correlation coefficient.

**Table 5:** **Correlations between RNA expression of CXCL1 and gene expression of TGF-**β **signaling components**

Signaling component	r	95% CI	***p***-value
*TGFB1*	0.19	-0.09 to 0.45	0.18
*TGFBR2*	0.12	-0.16 to 0.39	0.12
*SMAD2*	0.33	0.06 to 0.57	0.01
*SMAD3*	0.11	-0.17 to 0.38	0.42

Association between CXCL1 protein expression and expression of TGF-β signaling components was determined using Spearman Correlation analysis of IDC stroma and normal adjacent stroma.

Significance determined by *p*<0.05. r= correlation coefficient.

**Figure 6:**
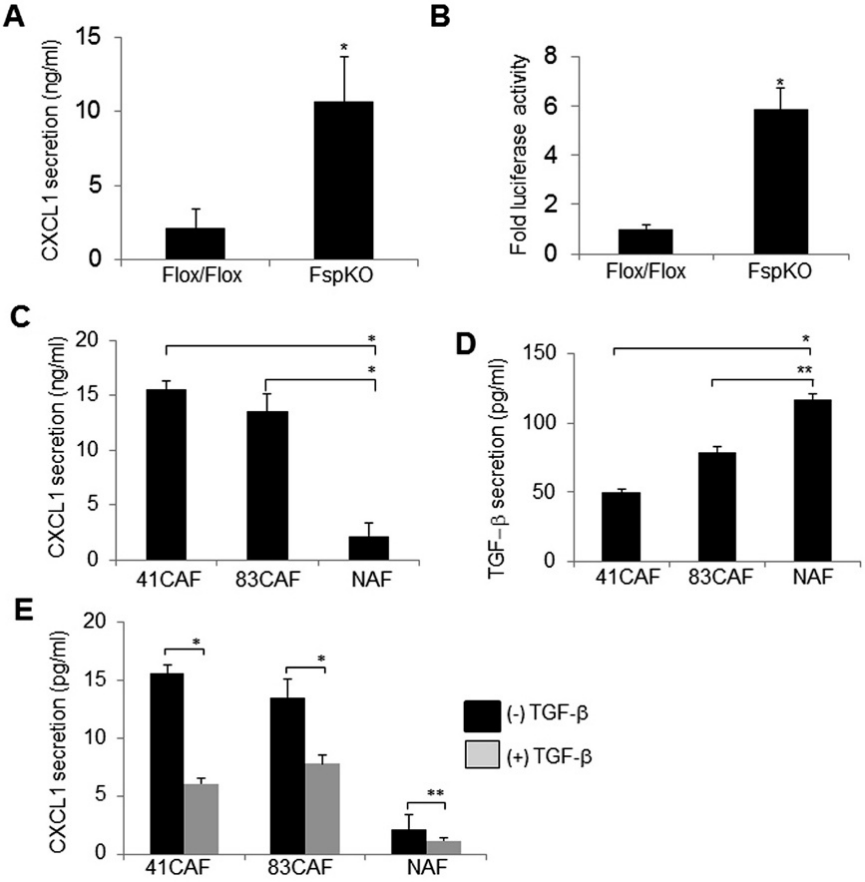
**CXCL1 expression is inversely associated with TGF-β signaling in mammary fibroblasts. A**. Conditioned medium from Flox/Flox or FspKO fibroblasts were analyzed for CXCL1 secretion by ELISA. **B**. Flox/Flox control or FspKO fibroblasts were co-transfected with CXCL1 firefly and Renilla luciferase reporter constructs and analyzed for luciferase activity. Values are normalized to Renilla. **C**.-D. Conditioned medium from carcinoma associated fibroblast cell lines (41CAF, 83CAF) or normal fibroblasts (NAF) were analyzed by ELISA for CXCL1 **(C)** or TGF-β secretion **(D)**. **E**. Fibroblasts were treated with 5 ng/ml TGF-β for 48 hours and analyzed for CXCL1 secretion by ELISA. Statistical analysis was determined by two-tailed Student t-tests. Statistical significance was determined by *p* <0.05. *p ≤0.001, **p <0.05. Values are expressed as Mean ± SEM. Experiments were conducted in triplicate with three replicate samples per group.

We performed further studies to clarify the role of TGF-β signaling on CXCL1 expression in fibroblasts. In previous studies, we had generated a conditional knockout mouse model (FspKO), in which exon 2 of the *Tgfbr2* gene was deleted by cre, placed under the control of the Fsp1 promoter. Mammary fibroblasts isolated from FspKO mice and control mice (Flox/Flox) were isolated and immortalized. Immortalized fibroblasts were shown to be genetically stable and behave similarly to primary fibroblasts in vitro and when transplanted into mice [49]. These studies demonstrate a reliable model to study the role of TGF-β signaling on CXCL1 expression in mammary fibroblasts. By ELISA, a significant increase in CXCL1 protein secretion was detected in FspKO fibroblasts, compared to control fibroblasts (Figure 6A). The increased protein secretion corresponded to elevated luciferase activity of the CXCL1 promoter in FspKO fibroblasts (Figure 6B). To determine whether CXCL1 expression levels in FspKO fibroblasts were representative of chemokine expression in CAFs, we analyzed for CXCL1 expression in mammary fibroblasts isolated from MMTV-PyVmT transgenic mice. CXCL1 expression was significantly higher in CAF cell lines compared to normal fibroblasts, and corresponded to lower levels of TGF-β expression in CAFs (Figures 6C-D). Furthermore, treatment of TGF-β inhibited CXCL1 secretion in the fibroblast cell lines (Figure 6E). These data demonstrate that TGF-β signaling negatively regulates expression of CXCL1 in CAFs.

## Discussion

Empirical studies in animal models and human tissues have established the importance of stromal fibroblasts on cancer progression [15, 64]. However, the concept of the “tumor promoting” fibroblast has not been clearly defined. While recent studies have shown that the CXCL1 chemokine is expressed in tumor epithelial cells and stromal cells, the relevance of stromal CXCL1 expression has remained poorly understood. Here we report that elevated CXCL1 expression in breast cancer stroma is associated with tumor recurrence and decreased patient survival. We also show that CXCL1 is localized to α-SMA and FSP1 expressing fibroblasts, and is negatively regulated by TGF-β signaling. These studies contribute to the definition of the tumor promoting fibroblast, identify similarities and differences in CXCL1 RNA and protein expression patterns, and demonstrate a clinical significance for CXCL1 expression in cancer stroma.

In order to overcome the challenges of collecting sufficient numbers of tissue samples, we used both commercial and institutional resources. These resources allowed us to collect the tissues needed to perform the immunohistochemistry staining and quantify the level of protein expression in the breast cancer stroma. One limitation to the immunohistochemistry analysis was that we were unable to determine an association between stromal CXCL1 protein expression and clinical outcome, due to lack of follow-up data from either sources. While we did not observe significant associations between stromal CXCL1 expression and age or ethnicity, we were unable to determine associations between stromal CXCL1 and other risk factors such as genetics, life-style or family history [65, 66]. The Finak microarray dataset provided new data demonstrating a clinical relevance for RNA expression of CXCL1 in the stroma. However, one limitation was that we were unable to determine the association between stromal CXCL1 RNA expression and prognostic factors such as biomarker expression or lymph node status, as these data were not provided with the Finak dataset. In addition, we were unable to determine an exact relationship between stromal CXCL1 RNA and protein expression, as these samples were not matched. To overcome these limitations, it would be of interest in the future to conduct studies using a sample size population with more complete clinical profiles that would enable us to match CXCL1 RNA expression with protein expression.

In our studies, we observed important similarities between stromal CXCL1 protein and RNA expression levels in breast stromal tissues. Intensity of RNA and protein expression levels was higher in breast tumors than in normal breast tissues. In particular, elevated expression levels of stromal CXCL1 RNA and protein were detected in high grade tumors, and there were no significant differences in association with the other prognostic factors examined. We also observed several differences in RNA and protein expression of CXCL1 in the breast stroma. Stromal CXCL1 protein expression was positively expressed in all tumors examined, while the RNA was expressed in a small subset of breast tumor samples. While stromal CXCL1 protein expression correlated with tumor grade, significant levels of stromal CXCL1 RNA expression was observed only in high grade tumors. In addition, the stromal CXCL1 protein expression inversely correlated with expression of TGF-β, phospho-SMAD3 and phospho-SMAD2. In contrast, CXCL1 RNA levels positively correlated with *SMAD2* gene expression. These differences in expression patterns for stromal CXCL1 are consistent with previous studies showing significant variations between RNA and protein levels observed in endometrial, colorectal and bladder carcinomas [67].

Multiple factors could account for differences in CXCL1 protein and RNA expression. RNA and protein expression data were from unmatched samples, and the heterogeneity of breast cancer patients could have contributed to differences in RNA and protein expression levels. It is also possible that post-transcriptional and post-translational mechanisms contribute to the differences in CXCL1 RNA and protein expression in breast cancer stroma in lower grade tumors. Studies have shown that NF-κB, PARP (poly ADP ribose polymerase) and CREB (cAMP Response Element Binding) proteins positively regulate CXCL1 transcription, while CAAT displacement proteins negatively regulate CXCL1 transcription. Their activities have been reported in breast cancer and could affect CXCL1 transcript levels [68, 69]. Post-transcriptional mechanisms active in breast cancer include microRNA activity [70]. Mir-7641 has been shown to regulate CXCL1 expression in endothelial cells [71]. Mir200 has been shown to modulate CXCL1 mRNA expression in invasive breast cancers [72]. It is possible that microRNA levels in breast tumor tissues may affect CXCL1 RNA levels. Possible post- translational mechanisms for CXCL1 involve biochemical binding between CXCL1 and heparin in the extracellular matrix to enhance CXCL1 protein half-life [73]. Thus, it is possible for stromal CXCL1 protein expression levels to be higher than RNA levels, as observed in breast cancer stroma.

Our studies indicate that CXCL1 is elevated in breast CAFs, and is associated with increased tumor recurrence and tumor grade. As the binding receptors CXCR1 and CXCR2 are expressed on myeloid derived cells and carcinoma cells [74, 75], CXCL1 expression in CAFs may serve to regulate paracrine signaling interactions with immune cells and cancer cells to promote chemo-resistance and tumor progression. This hypothesis is supported by previous studies on CXCL1 expression in the MMTV-PyVmT transgenic mouse model, where CXCL1 functioned to recruit myeloid immune suppressor cells that enhanced survival and invasion of mammary tumors. Treatment of mammary tumors with Doxorubicin resulted in the selection of drug resistant mammary carcinoma cells with elevated CXCL1 expression in cancer cells [54]. Studies have shown that chemotherapies do not efficiently target CAFs for cell death but rather enhance the tumor promoting activities of fibroblasts by promoting secretion of growth factors and cytokines [24, 25]. It is possible that CXCL1 expression in CAFs is retained or further elevated after chemotherapy treatment, serving to promote the survival and selection of chemo-resistant tumor cells. It would be of interest to conduct further studies on stromal CXCL1 expression on breast tumor tissues from patients treated with chemotherapies, and conduct functional studies in animal models. These studies would clarify the role of CAF-derived CXCL1 on breast cancer progression and tumor recurrence.

Our studies introduce new findings that elevated CXCL1 expression in breast cancer stroma inversely correlate with expression of TGF-β signaling components. Furthermore, we find that TGF-β suppresses CXCL1 expression in cultured CAFs. These studies indicate that CAFs decrease TGF-β signaling to enhance breast cancer progression, partly by increasing CXCL1 chemokine expression. These observations are consistent with previous studies demonstrating a tumor suppressive role for TGF-β signaling in the breast stroma. Transgenic mice expressing dominant negative TGF-β type II receptor in mammary stroma exhibited mammary hyperplasia [76]. Cre mediated deletion of exon 2 of TGF-β type II receptor gene (*Tgfbr2*) in mammary fibroblasts (FspKO) inhibited TGF-β mediated suppression of fibroblast proliferation. Co-transplantation of FspKO fibroblasts with 4 T1 and PyVmT mammary carcinoma cells in the subrenal capsule of nude mice enhanced tumor progression. These tumor promoting phenotypes were associated increased expression of growth factors and receptor tyrosine kinases [49, 77, 78]. It is possible that increased CXCL1 expression would act in concert with increased growth factor expression to enhance invasiveness of breast carcinomas.

We and others observed expression of TGF-β and phosphorylated Smad proteins in cancer cells indicating active TGF-β signaling. As TGF-β is expressed in the epithelium and could signal to fibroblasts in a paracrine manner [79–82], it is unclear how TGF-β signaling would be down-regulated in the stroma. As fibroblasts are more genetically stable than cancer cells [83], it is possible that mechanisms other than genetic mutations would down-regulate TGF-β signaling in CAFs. Stat3, MAPK and NF-κB inhibit TGF-β signaling in cells [84, 85], and may contribute to decreased TGF-β expression and phosphorylated SMAD2 or SMAD3 expression in breast CAFs. It is also possible that epigenetic mechanisms, such as methylation of *TGFB* and *SMAD* promoters [86, 87] would silence gene expression and down-regulate TGF-β signaling in breast cancer stroma. It would be of interest to further study how TGF-β signaling is regulated in the context of breast stromal tissues, in order to better understand how CAFs are regulated.

## Conclusions

In summary, we provide insight into the clinical significance of stromal derived CXCL1 expression, and show that α-SMA and FSP1 positive CAFs in breast cancer stroma are sources of CXCL1 expression. In addition, we also demonstrate that the TGF-β signaling pathway is an important negative regulator CXCL1 expression in breast CAFs. As CXCL1 is increasingly shown to play important roles in tumor recurrence and chemoresistance, further studies on the impact of CXCL1 expression on the breast tumor microenvironment will aid in the development of novel anti-cancer therapies to combat drug resistant tumors.

## Electronic supplementary material

Additional file 1: Figure S1: Expression of stromal CXCL1 in DCIS subtypes. DCIS patient specimens were immunostained for CXCL1 protein expression and quantified for expression in the stroma among the different classified subtypes. Subtypes are organized in descending order of diagnosis. Statistical analysis among groups was performed using the Kruskall-Wallis test followed by Dunn’s post-*hoc* comparison. Statistical significance was determined by *p* <0.05. ***p ≥0.05, in comparison with all groups. Values are expressed as Mean ± SEM. (JPEG 130 KB)

Additional file 2: Figure S2: Expression of stromal CXCL1 in IDC subtypes. IDC patient specimens were immunostained for CXCL1 and quantified for expression in the stroma among the different classified subtypes. Subtypes are organized in descending order of diagnosis. Statistical analysis among groups was performed using the Kruskall-Wallis test followed by Dunn’s post-*hoc* comparison. Statistical significance was determined by *p* <0.05. NOS = Not Otherwise Specified. ***p ≥0.05, in comparison with all groups. Values are expressed as Mean ± SEM. (JPEG 134 KB)

Additional file 3: Table S1: Relationship between age and CXCL1 expression in breast cancer stroma. The association between age and stromal CXCL1 protein expression was determined in the US Biomax and BCRF datasets by Spearman Correlation analysis. Significance determined by *p* < 0.05. r = correlation coefficient. (CSV 102 bytes)

Additional file 4: Figure S3: Expression of stromal CXCL1 in individual datasets. Levels of stromal CXCL1 were compared between the US Biomax and BCRF datasets. Statistical analysis among groups was performed using the Kruskall-Wallis test followed by Dunn’s post-*hoc* comparison. Statistical significance was determined by *p* <0.05. ***p ≥0.05, in comparison with all groups. Mean ± SEM is shown for each group. (JPEG 80 KB)

Additional file 5: Figure S4: Stromal CXCL1 expression is not associated with grade of DCIS. DCIS patient specimens were immunostained for CXCL1 protein expression and analyzed for association with histologic grade. *n* =2 for DCIS grade 1, *n* =7 for DCIS grade 2 and *n* =13 for DCIS grade 3. Statistical analysis among groups was performed using the Kruskall-Wallis test. Statistical significance was determined by *p* <0.05. ***p ≥0.05, in comparison with all groups. Values are expressed as Mean ± SEM. (JPEG 76 KB)

## Abbreviations

IDC: Invasive ductal carcinoma
DCIS: Ductal Carcinoma In Situ
CAFs: Carcinoma associated fibroblasts
α-sma: Alpha smooth muscle actin
Fsp1: Fibroblast Specific Protein 1
FAP: Fibroblast activating protein
FspKO: Fibroblast specific protein knockout for the Tgfbr2 gene
TGF-β: Transforming growth factor beta
TGFBR2: TGF-β type II Receptor gene
Smad: Combination of the C. elegans gene Sma and drosophila gene mothers against decapentaplegic
BRCF: Biospecimen repository core facility
TMA: Tissue microarray
DAB: 3,3′-Diaminobenzidine
MMTV: Mouse mammary tumor virus
PyVmT: Polyoma middle T
ELISA: Enzyme linked immuno-sorbent assay
NOS: Not otherwise specified
CREB: cAMP response element-binding
NF-κB: Nuclear factor Kappa B
PARP: Poly ADP ribose polymerase.
