# Supplementary figures and images for: Elevated CXCL1 expression in breast cancer stroma predicts poor prognosis and is inversely associated with expression of TGF-β signaling proteins
Source: BMC Cancer. 2014 Oct 24;14:781. doi: 10.1186/1471-2407-14-781 (PMC4221705; doi:10.1186/1471-2407-14-781)

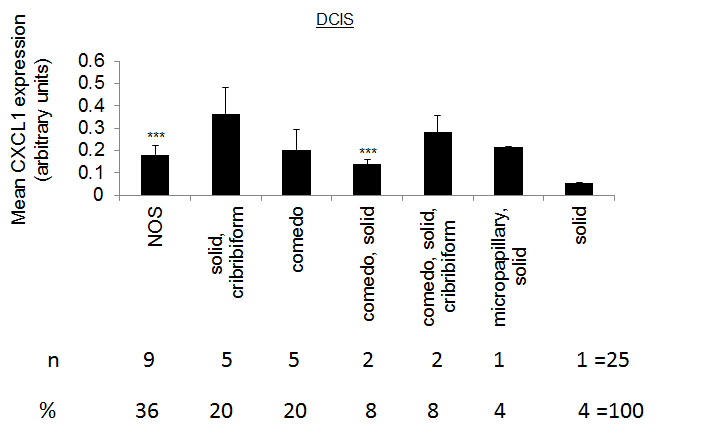

Supplement: Supplementary file 1 — Additional file 1: Figure S1: Expression of stromal CXCL1 in DCIS subtypes. DCIS patient specimens were immunostained for CXCL1 protein expression and quantified for expression in the stroma among the different classified subtypes. Subtypes are organized in descending order of diagnosis. Statistical analysis among groups was performed using the Kruskall-Wallis test followed by Dunn’s post-hoc comparison. Statistical significance was determined by p <0.05. ***p ≥0.05, in comparison with all groups. Values are expressed as Mean ± SEM. (JPEG 130 KB) [file 12885_2014_4963_MOESM1_ESM.jpeg]

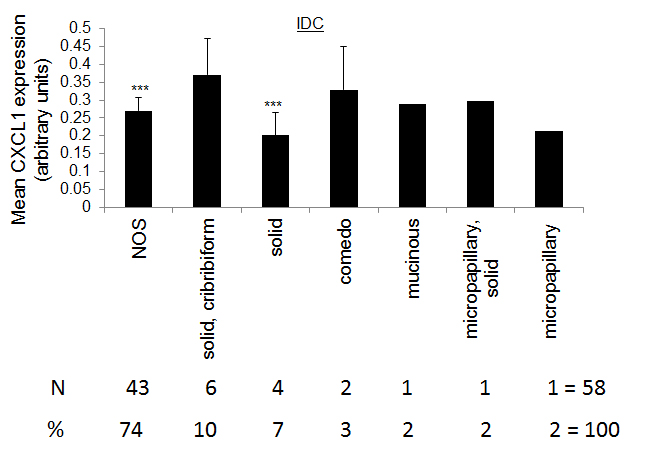

Supplement: Supplementary file 2 — Additional file 2: Figure S2: Expression of stromal CXCL1 in IDC subtypes. IDC patient specimens were immunostained for CXCL1 and quantified for expression in the stroma among the different classified subtypes. Subtypes are organized in descending order of diagnosis. Statistical analysis among groups was performed using the Kruskall-Wallis test followed by Dunn’s post-hoc comparison. Statistical significance was determined by p <0.05. NOS = Not Otherwise Specified. ***p ≥0.05, in comparison with all groups. Values are expressed as Mean ± SEM. (JPEG 134 KB) [file 12885_2014_4963_MOESM2_ESM.jpeg]

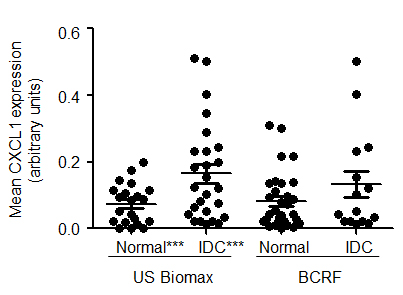

Supplement: Supplementary file 4 — Additional file 4: Figure S3: Expression of stromal CXCL1 in individual datasets. Levels of stromal CXCL1 were compared between the US Biomax and BCRF datasets. Statistical analysis among groups was performed using the Kruskall-Wallis test followed by Dunn’s post-hoc comparison. Statistical significance was determined by p <0.05. ***p ≥0.05, in comparison with all groups. Mean ± SEM is shown for each group. (JPEG 80 KB) [file 12885_2014_4963_MOESM4_ESM.jpeg]

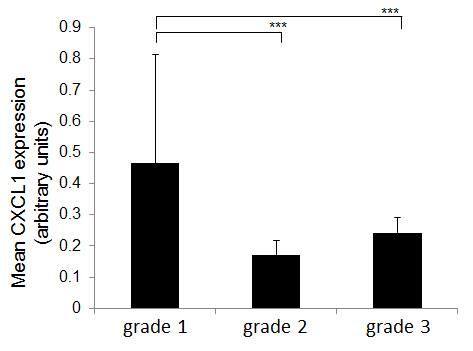

Supplement: Supplementary file 5 — Additional file 5: Figure S4: Stromal CXCL1 expression is not associated with grade of DCIS. DCIS patient specimens were immunostained for CXCL1 protein expression and analyzed for association with histologic grade. n =2 for DCIS grade 1, n =7 for DCIS grade 2 and n =13 for DCIS grade 3. Statistical analysis among groups was performed using the Kruskall-Wallis test. Statistical significance was determined by p <0.05. ***p ≥0.05, in comparison with all groups. Values are expressed as Mean ± SEM. (JPEG 76 KB) [file 12885_2014_4963_MOESM5_ESM.jpeg]
